# Comparative analysis of the JAK/STAT signaling through erythropoietin receptor and thrombopoietin receptor using a systems approach

**DOI:** 10.1186/1471-2105-10-S1-S53

**Published:** 2009-01-30

**Authors:** Hong-Hee Won, Inho Park, Eunjung Lee, Jong-Won Kim, Doheon Lee

**Affiliations:** 1 Department of Bio and Brain Engineering, Korea Advanced Institute of Science and Technology, 373-1 Guseong-dong, Yuseong-gu, Daejeon 305-710, South Korea; 2 Samsung Biomedical Research Institute, Samsung Medical Center, 50 Ilwon-dong, Gangnam-gu, Seoul 135-710, South Korea; 3 Department of Laboratory Medicine and Genetics, Sungkyunkwan University School of Medicine, Samsung Medical Center, 50 Ilwon-dong, Gangnam-gu, Seoul 135-710, South Korea

## Abstract

**Background:**

The Janus kinase-signal transducer and activator of transcription (JAK/STAT) pathway is one of the most important targets for myeloproliferative disorder (MPD). Although several efforts toward modeling the pathway using systems biology have been successful, the pathway was not fully investigated in regard to understanding pathological context and to model receptor kinetics and mutation effects.

**Results:**

We have performed modeling and simulation studies of the JAK/STAT pathway, including the kinetics of two associated receptors (the erythropoietin receptor and thrombopoietin receptor) with the wild type and a recently reported mutation (JAK2V617F) of the JAK2 protein.

**Conclusion:**

We found that the different kinetics of those two receptors might be important factors that affect the sensitivity of JAK/STAT signaling to the mutation effect. In addition, our simulation results support clinically observed pathological differences between the two subtypes of MPD with respect to the JAK2V617F mutation.

## Background

The Janus kinase-signal transducer and activator of transcription (JAK/STAT) pathway has been frequently reported to be responsible for oncogenic processes such as uncontrollable proliferation, apoptosis resistance, sustained angiogenesis, and immune evasion [1]. Recently, several research groups have demonstrated the existence of a point mutation in JAK2 in most patients with polycythaemia vera (PV), and approximately half of patients with essential thrombocythaemia (ET) and primary myelofibrosis (PMF) which are subtypes of myeloproliferative disorder (MPD) [2-4]. The mutation results in a substitution of valine for phenylalanine at codon 617 of JAK2 (JAK2V617F) and leads to cytokine hypersensitivity and cytokine independent activation of the JAK/STAT pathway. As a consequence, the expression of JAK2V617F causes the production of hematopoietic colonies that are characteristic of MPD patients.

Although JAK2V617F is well known to be an important marker for MPD, it has also been reported that the frequencies of the JAK2V617F allele in PV, ET, and PMF patients are significantly different. The frequency of the JAK2V617F allele in each MPD subtype is shown in Table 1. Moreover, homozygous JAK2V617F mutant erythroid colonies can be grown from >30% of patients with PV, while it is rarely observed in patients with ET (Table 1) [5]. Hematopoietic colonies grown from ET patients mostly have wild type JAK2 or heterozygous JAK2 proteins. These findings imply that there are important genetic differences between PV and ET in terms of JAK/STAT signaling. Erythropoietin (Epo) and thrombopoietin (Tpo) induce JAK/STAT signaling through the corresponding receptors, the erythropoietin receptor (EpoR) and thrombopoietin receptor (TpoR), and regulate the production of red blood cells and platelets, respectively. Thus, it has been suggested that JAK/STAT signaling through EpoR and TpoR are related to PV and ET, respectively [4].

**Table 1:** Frequency and mutational state of the JAK2V617F allele in MPD patients.

		Mutational state^b^		
		
Disease	Frequency^a^	WT	Hetero	Homo
PV	81–99%	-	68%	32%
ET	41–72%	40%	58%	2%
PMF	39–57%	-	-	-

^a^Adapted from Levine et al. [4 ].^b^Adapted from Vannucchi et al. [5].

Furthermore, a dosage effect of JAK2V617F on JAK/STAT signaling has been reported. Constitutive activation of JAK/STAT signaling observed in cells transfected with only the JAK2V617F gene were diminished when cells were transfected with both the wild type and JAK2V617F gene [2].

Although several efforts have been made for modeling the JAK/STAT pathway [6-10], to the best of our knowledge, there has been no comparative analysis performed for JAK/STAT signaling through EpoR and TpoR that is related to specific disease phenotypes. In this work, we aimed to understand the role of the JAK2V617F mutation in the formation of the disease phenotypes, PV and ET, through systems analysis. The main question of this work is how the two cytokine ligands, Epo and Tpo, and the corresponding receptors are related to PV and ET in regard to the activation of the JAK/STAT pathway. To study the different dynamic characteristics, we considered not only the kinetics of the receptors but also the relationship of the receptors with JAK2. In addition, the behavior of the suppressor of cytokine signaling (SOCS) protein, a negative regulator of JAK/STAT signaling, was investigated.

Through this systems approach, we found that the JAK2V617F mutation led to the over-activation of JAK/STAT signaling and the effect of the mutation was different between PV and ET based on the different receptor kinetics, supporting the different mutation patterns clinically observed in the patients.

## Results and Discussion

### Comparison of wild type JAK/STAT signaling through EpoR and TpoR

**Figure 1:**
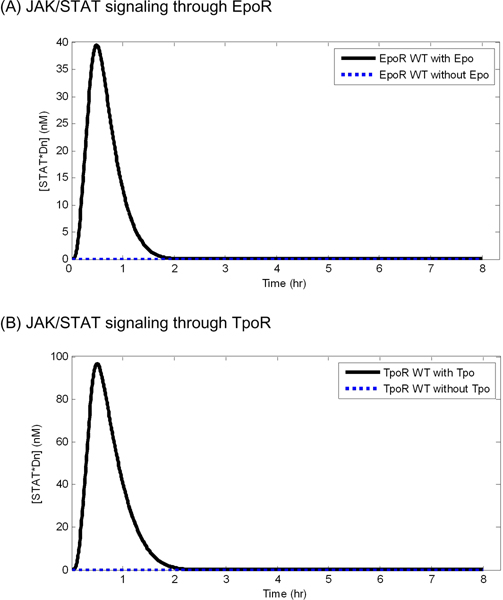
**The simulated concentration of nuclear phosphorylated STAT dimers with or without over 8 hour continuous exposure to the cytokine ligands (Epo and Tpo) in wild type models**. The black solid curve indicates simulated concentration with ligand exposure and the blue dashed curve indicates simulated concentration without ligand exposure.

In the absence of continuous exposure of cytokine ligands, both EpoR and TpoR did not transduce a signal through the JAK/STAT pathway in wild type models, and accordingly, the concentration of nuclear phosphorylated STAT dimers (STAT*Dn) did not change (Figure 1). The binding of cytokine to the receptors led to signal transduction and the concentration of STAT*Dn reached a peak within 1 hour and reverted to its initial concentration, suggesting that the negative regulators control the pathway, regardless of receptor dynamics. Compared with EpoR with a high disappearance rate (k_d_ = 0.0452), TpoR with a relatively low disappearance rate (k_d_ = 0.0152) showed a stronger effect on the level of peak concentration of STAT*Dn. The peak concentration of STAT*Dn was increased by the signal transduction through TpoR approximately 2~3 times as compared to STAT*Dn that was increased by signal transduction through EpoR.

### Comparison of JAK/STAT signaling with wild type JAK2 and JAK2V617F mutant proteins

**Figure 2:**
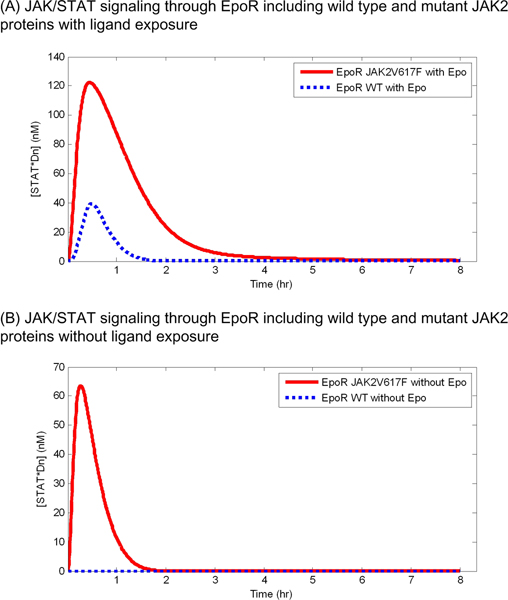
**JAK/STAT signaling through EpoR including wild type and mutant JAK2 (JAK2V617F) proteins**. The simulated concentrations of  nuclear phosphorylated STAT dimers with and without continuous Epo exposure are shown in (A) and (B), respectively.

As represented in Figure 2, with 8 hour continuous exposure to Epo, the JAK2V617F mutant model shows a higher level of STAT*Dn than the wild type model. The peak concentration of STAT*Dn in the mutant model increased approximately 80 nM more than that in the wild type model, and the concentration of STAT*Dn in the mutant model decreased slowly as compared to that of the wild type model (Figure 2A). The concentration of STAT*Dn reverted to its initial concentration within 2 hours in the wild type model while it reverted after more than 4 hours in the mutant model. This observation indicates that the negative regulator SOCS could not control JAK/STAT signaling with the JAK2V617F mutant as effectively as with the wild type model [11]. As similar to continuous Epo exposure, of particular importance, the JAK2V617F mutant model shows a high peak concentration even in the absence of Epo (Figure 2B). This result is consent with the known pathological condition observed in patients with PV such that STAT*Dn is highly activated even without ligand exposure [2].

**Figure 3:**
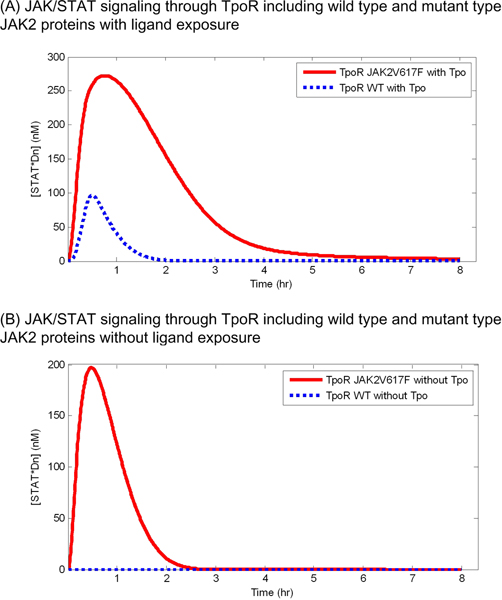
**JAK/STAT signaling through TpoR including wild type and mutant JAK2 (JAK2V617F) proteins**. The simulated concentrations of nuclear phosphorylated STAT dimers with and without continuous Tpo exposure are shown in (A) and (B), respectively.

The JAK2V617F mutant JAK/STAT signaling through TpoR also showed an increased level of the concentration of STAT*Dn in both cases with and without continuous exposure to Tpo (Figure 3). However, the increased level of STAT*Dn induced by the mutant model in JAK/STAT signaling through TpoR was higher than that in the signaling through EpoR. Whereas the concentration of STAT*Dn in the wild type model decreased completely to its initial concentration within 2 hours, the concentration in the TpoR JAK2V617F mutant model was kept high for more than 4 hours (Figure 3A). The TpoR JAK2V617F mutant model was shown to increase the concentration of STAT*Dn greatly (Figure 3B). The increased level and duration of high concentration of STAT*Dn became larger in the TpoR JAK2V617F mutant model than in the EpoR JAK2V617F mutant model, indicating that the difference in the receptor kinetics between the EpoR and TpoR model can affect the response of the JAK2V617F mutation on the pathway.

### Comparison of JAK/STAT signaling with JAK2V617F through EpoR and TpoR

**Figure 4:**
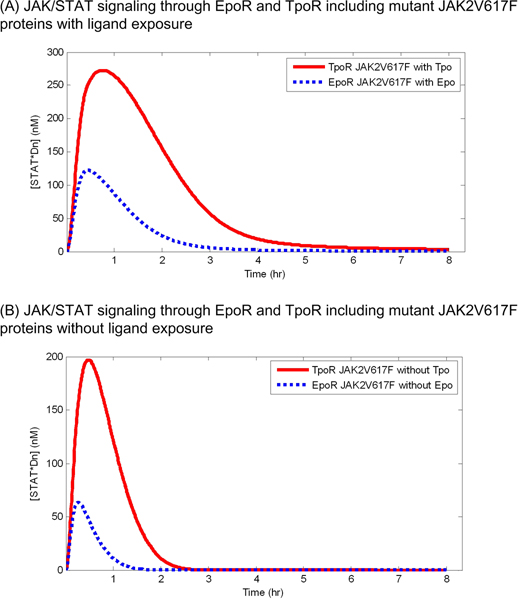
**Effects of the JAK2V617F mutation on JAK/STAT signaling through EpoR and TpoR**. The simulated concentrations of nuclear phosphorylated STAT dimers are presented in the solid curve (signaling through TpoR) and in the dashed curve (signaling through EpoR).

Figure 4 shows a comparison of the effects of the JAK2V617F mutation on JAK/STAT signaling through EpoR and TpoR. The peak concentration of STAT*Dn induced by signaling through TpoR was much higher than that through EpoR (Figure 4A). Moreover, the duration of the high concentration of STAT*Dn was longer in signaling through TpoR than that through EpoR. This observation was also found in the case without continuous ligand exposure. In the absence of ligand, signaling through TpoR produced approximately 130 nM of STAT*Dn more than signaling through EpoR (Figure 4B).

**Figure 5:**
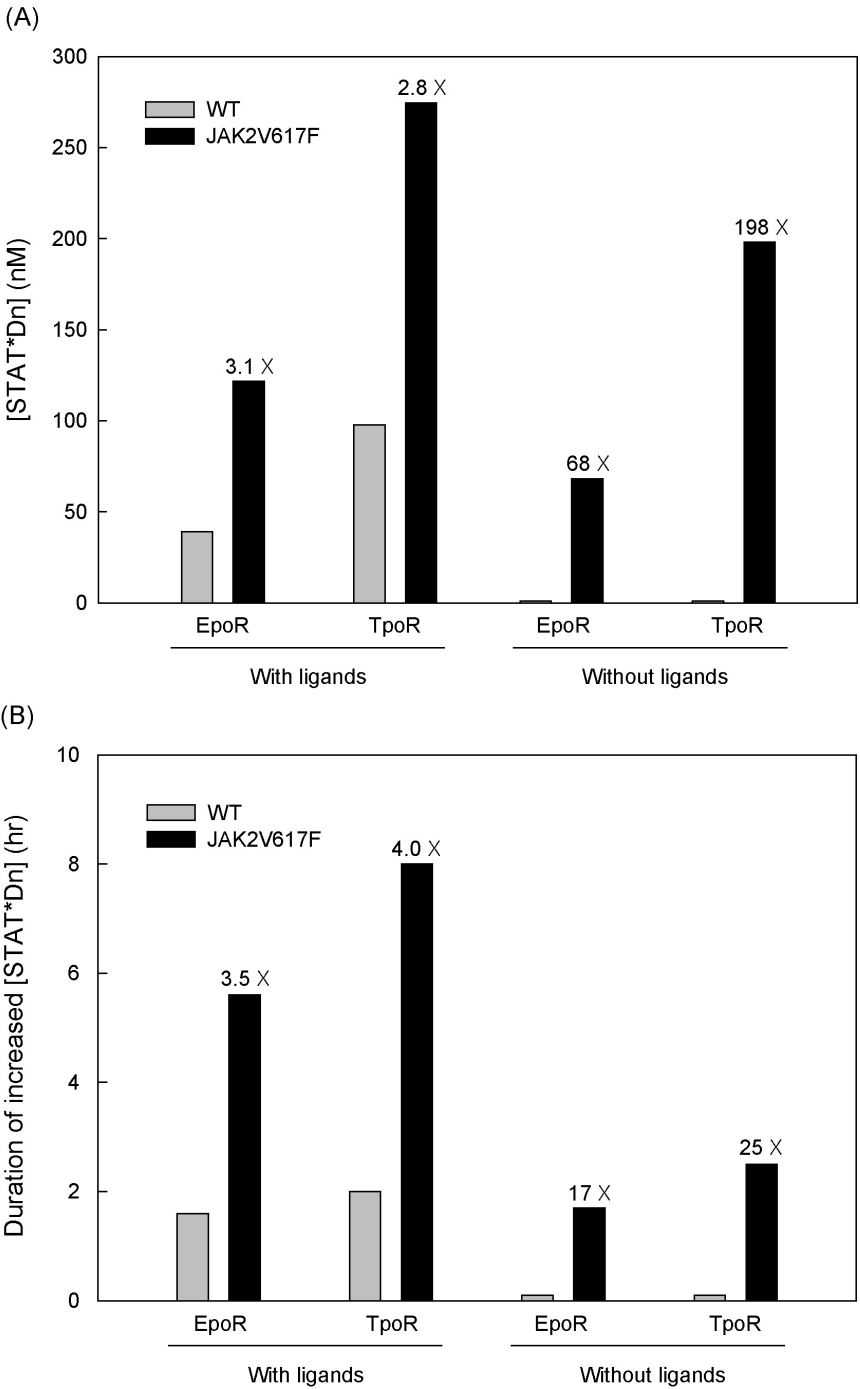
**The increased peak concentration (A) and the signal duration (B) of nuclear phosphorylated STAT dimers in the model with JAK2V617F proteins**. The gray and black bars represent the wild type and JAK2V617F mutant model, and the numbers on the black bars denote the ratio of the concentration of [STAT*Dn] in the model with JAK2V617F to the model with the wild type JAK2 protein. We assumed [STAT*Dn] was 1 nM for the model with wild type JAK2 without ligands and the continuance duration was 0.1 hour to calculate fold changes in the model with the mutant JAK2V617F.

Figure 5 illustrates the peak intensity and the duration of JAK/STAT signaling through EpoR and TpoR in models including wild type JAK2 or mutant JAK2 proteins (JAK2V617F) under ligand exposure or no ligand exposure. With ligand exposure, JAK/STAT signaling with the JAK2V617F mutation through EpoR and TpoR increased similarly, 3.1 and 2.8 times as strong in intensity, and 3.5 and 4.0 times as long in duration as with wild type JAK2. However, without ligand exposure, which is an important condition in terms of MPD pathology, there is virtually no JAK/STAT signaling in the model with wild type JAK2, but JAK/STAT signaling with the JAK2V617F mutant protein showed a signifiacant increase in both intensity and duration. Interestingly, the amount of signaling increase through TpoR is much larger than that through EpoR (198 versus 68 in intensity and 25 versus 17 in duration).

These findings suggesting that JAK/STAT signaling through TpoR is more sensitive to the mutation effect than through EpoR support that a lower frequency of the JAK2V617F mutation is observed in ET patients than in PV patients. JAK/STAT signaling through EpoR is considered as mainly related with the production of red blood cells, whose excessive increase is an important clinical characteristic of PV patients, and signaling through TpoR is related with the production of platelets whose excessive production is a manifestation of ET. In addition, the findings also support the rare homozygosity of JAK2V617F in ET patients while a significant population of PV patients carry homozygous JAK2V617F alleles.

## Conclusion

This study has shown that a systems approach can be useful for a comparative analysis of receptor kinetics of a signal transduction pathway in the context of the pathological domain for understanding MPD, especially JAK/STAT signaling through EpoR and TpoR. The simulated results indicate that the difference of receptor kinetics between Epo-induced and Tpo-induced JAK/STAT pathway might affect the sensitivity of the pathway to JAK2V617F, a gain-of-function mutation. Particularly, in the absence of ligands (this condition might be critical in chronic diseases as well as for MPD), JAK/STAT signaling through TpoR with a higher cell surface expression and a lower disappearance rate was more sensitive to the mutation effect than through EpoR. Accordingly, the peak and steady state concentration level of STAT*Dn, regarded as an indicator of the amount of JAK/STAT signaling, was set to an abnormally high level by the mutant model of JAK/STAT signaling through TpoR. These observations can be in accord with the clinical observation of the rarer homozygosity of JAK2V617F in ET patients than in PV patients. The effect of the mutation in PV is relatively weak and the high rate of homozygosity or dosage of mutant JAK2 proteins might be required to induce the PV phenotype [12,13].

In future work, to investigate the pathway with regard to the pathogenesis of PV and ET, the model needs to be extended further to include some proteins related with disease phenotypes. Since PV patients can be characterized by an abnormal increase in the number of three types of blood cells – erythrocytes, granulocytes, and platelets [14] – the model for PV should involve hematopoietic transcription factors such as GATA-1, GATA-2 [15,16], C/EBPε and Fli-1 [17,18] that correspond to each lineage. In addition, some previous studies have shown that the cytokine receptors are also needed for JAK2V617F-mediated transformation [19,20]. Therefore, it might be informative to include JAK as a chaperone for the receptor kinetics into the model.

## Methods

### JAK/STAT pathway model

**Figure 6:**
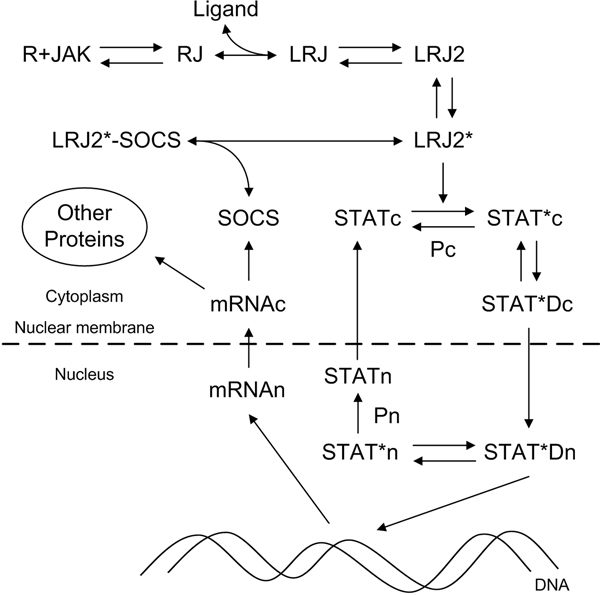
**The JAK/STAT pathway**. Each node indicates the component involving the transduction signaling or negative regulation of the pathway (R: receptor, JAK: JAK2, RJ: receptor-JAK2 complex, Ligand: Epo or Tpo, LRJ: ligand bound RJ, Pc: cytoplasmic phosphatase, Pn: nuclear phosphatase, SOCS: negative regulator). * and D denote a phosphorylation and a dimerization, respectively. According to the location in a cell, each molecule is tagged by c (cytoplasm) or n (nucleus). The system output was calculated by the level of nuclear phosphorylated STAT dimers (STAT*Dn).

We adopted the JAK/STAT pathway model by Yamada et al. [7]. The schematic diagram (Figure 6) represents the cytokine ligand (Epo or Tpo) induced JAK/STAT pathway. The binding of ligand to the Receptor-JAK complex (RJ) results in the dimerization of LRJ. The dimerized LRJ (LRJ2) is phosphorylated by JAK and the phosphorylated LRJ dimer (LRJ2*) phosphorylates STAT in the cytoplasm (STATc). The phosphorylated STAT (STAT*c) dimerizes (STAT*Dc) and translocates to the nucleus (STAT*Dn), which functions as a transcription factor for various genes. One of the genes induced by STAT*Dn is SOCS that inhibits the kinase activity of LRJ2*. STAT*Dn also induces other proteins related to the cell proliferation of erythroid, myeloid, and megakaryocytic lineage. The level of nuclear phosphorylated STAT dimers (STAT*Dn) was considered as the output of the pathway. We performed simulations using the Systems Biology Toolbox [21] with Matlab (The Mathworks, Natick, MA USA).

### Receptor kinetics

To understand the dynamic differences of the Epo and Tpo induced JAK/STAT pathway, we included receptor kinetics. Several studies have demonstrated that in the absence of JAK, a much larger fraction of the thrombopoietin receptor (TpoR) (~5 times) than the erythropoietin receptor (EpoR) is on the cell surface [22]. The studies have also shown that TpoR is rapidly internalized from the cell surface [23], and that Epo induces the rapid internalization and degradation of Epo while Tpo induces long-lasting signaling in target cells [24]. These observations may indicate that TpoR has a lower degradation rate than EpoR, which leads to the rapid recycling of TpoR although EpoR and TpoR have a similar rate of internalization. From previously reported experimental results [22,23], we estimated the disappearance rate (k_d_) of EpoR and TpoR, which comprises the internalization and degradation rate for the sake of simplicity. It was estimated that EpoR disappears approximately three times faster than TpoR. The estimated relative surface expression levels and disappearance rates are summarized in Table 2.

**Table 2:** Receptor kinetics constants.

	EpoR		TpoR	
	
	With Epo	Without Epo	With Tpo	Without Tpo
Relative surface expression [24]	12	12	60	60
Disappearance rate of receptor	0.0452	0.0038	0.0152	0.0013

### JAK2V617F mutant model

JAK2V617F mutant kinases bind to cytokine receptors and are phosphorylated in the absence of ligands, leading to the ligand-independent activation of downstream signaling pathways [4]. We assumed that the phosphorylation rate of cytoplasmic STAT depended on the concentration of the receptor-JAK complex (RJ) as well as active ligand binding receptors, as JAK2V617F is a gain-of-function mutation independent of ligand binding or negative regulation. The chemical reaction by which JAK2V617F was added to the wild type model is as follows:

d/dt(STAT*c) = WT reactions + k_JAK2V617F_ × RJ

The rate constant k_JAK2V617F_ was estimated based on reported experimental results [2].

## Competing interests

The authors declare that they have no competing interests.

## Authors' contributions

HW developed the idea, performed the modeling and simulations, and wrote the manuscript. IP and EL developed the idea and designed the experiments. EL revised the manuscript. JK and DL designed the overall research plan and edited the manuscript. All authors read and approved the manuscript.

## References

[B1] Yu H, Jove R (2004). The STATs of cancer – new molecular targets come of age. Nat Rev Cancer.

[B2] James C, Ugo V, Le Couedic JP, Staerk J, Delhommeau F, Lacout C, Garcon L, Raslova H, Berger R, Bennaceur-Griscelli A (2005). A unique clonal JAK2 mutation leading to constitutive signalling causes polycythaemia vera. Nature.

[B3] Baxter EJ, Scott LM, Campbell PJ, East C, Fourouclas N, Swanton S, Vassiliou GS, Bench AJ, Boyd EM, Curtin N (2005). Acquired mutation of the tyrosine kinase JAK2 in human myeloproliferative disorders. Lancet.

[B4] Levine RL, Pardanani A, Tefferi A, Gilliland DG (2007). Role of JAK2 in the pathogenesis and therapy of myeloproliferative disorders. Nat Rev Cancer.

[B5] Vannucchi AM, Antonioli E, Guglielmelli P, Rambaldi A, Barosi G, Marchioli R, Marfisi RM, Finazzi G, Guerini V, Fabris F (2007). Clinical profile of homozygous JAK2 617V>F mutation in patients with polycythemia vera or essential thrombocythemia. Blood.

[B6] Shudo E, Yang J, Yoshimura A, Iwasa Y (2007). Robustness of the signal transduction system of the mammalian JAK/STAT pathway and dimerization steps. J Theor Biol.

[B7] Yamada S, Shiono S, Joo A, Yoshimura A (2003). Control mechanism of JAK/STAT signal transduction pathway. FEBS Lett.

[B8] Papin JA, Palsson BO (2004). The JAK-STAT signaling network in the human B-cell: an extreme signaling pathway analysis. Biophysical Journal.

[B9] Zi Z, Cho KH, Sung MH, Xia X, Zheng J, Sun Z (2005). In silico identification of the key components and steps in IFN-gamma induced JAK-STAT signaling pathway. FEBS Lett.

[B10] Soebiyanto RP, Sreenath SN, Qu CK, Loparo KA, Bunting KD (2007). Complex systems biology approach to understanding coordination of JAK-STAT signaling. Biosystems.

[B11] Hookham MB, Elliott J, Suessmuth Y, Staerk J, Ward AC, Vainchenker W, Percy MJ, McMullin MF, Constantinescu SN, Johnston JA (2007). The myeloproliferative disorder-associated JAK2 V617F mutant escapes negative regulation by suppressor of cytokine signaling 3. Blood.

[B12] Hammond E, Shaw K, Carnley B, P'ng S, James I, Herrmann R (2007). Quantitative determination of JAK2 V617F by TaqMan: An absolute measure of averaged copies per cell that may be associated with the different types of myeloproliferative disorders. J Mol Diagn.

[B13] Tiedt R, Hao-Shen H, Sobas MA, Looser R, Dirnhofer S, Schwaller J, Skoda RC (2008). Ratio of mutant JAK2-V617F to wild-type Jak2 determines the MPD phenotypes in transgenic mice. Blood.

[B14] Ishii T, Zhao Y, Shi J, Sozer S, Hoffman R, Xu M (2007). T cells from patients with polycythemia vera elaborate growth factors which contribute to endogenous erythroid and megakaryocyte colony formation. Leukemia.

[B15] Shimamoto T, Ohyashiki K, Ohyashiki JH, Kawakubo K, Fujimura T, Iwama H, Nakazawa S, Toyama K (1995). The expression pattern of erythrocyte/megakaryocyte-related transcription factors GATA-1 and the stem cell leukemia gene correlates with hematopoietic differentiation and is associated with outcome of acute myeloid leukemia. Blood.

[B16] Persons DA, Allay JA, Allay ER, Ashmun RA, Orlic D, Jane SM, Cunningham JM, Nienhuis AW (1999). Enforced expression of the GATA-2 transcription factor blocks normal hematopoiesis. Blood.

[B17] Gombart AF, Kwok SH, Anderson KL, Yamaguchi Y, Torbett BE, Koeffler HP (2003). Regulation of neutrophil and eosinophil secondary granule gene expression by transcription factors C/EBP epsilon and PU.1. Blood.

[B18] Hart A, Melet F, Grossfeld P, Chien K, Jones C, Tunnacliffe A, Favier R, Bernstein A (2000). Fli-1 is required for murine vascular and megakaryocytic development and is hemizygously deleted in patients with thrombocytopenia. Immunity.

[B19] Huang LJ, Constantinescu SN, Lodish HF (2001). The N-terminal domain of Janus kinase 2 is required for Golgi processing and cell surface expression of erythropoietin receptor. Mol Cell.

[B20] Lu X, Levine R, Tong W, Wernig G, Pikman Y, Zarnegar S, Gilliland DG, Lodish H (2005). Expression of a homodimeric type I cytokine receptor is required for JAK2V617F-mediated transformation. Proc Natl Acad Sci USA.

[B21] Schmidt H, Jirstrand M (2006). Systems Biology Toolbox for MATLAB: a computational platform for research in systems biology. Bioinformatics (Oxford, England).

[B22] Walrafen P, Verdier F, Kadri Z, Chretien S, Lacombe C, Mayeux P (2005). Both proteasomes and lysosomes degrade the activated erythropoietin receptor. Blood.

[B23] Dahlen DD, Broudy VC, Drachman JG (2003). Internalization of the thrombopoietin receptor is regulated by 2 cytoplasmic motifs. Blood.

[B24] Tong W, Sulahian R, Gross AW, Hendon N, Lodish HF, Huang LJ (2006). The membrane-proximal region of the thrombopoietin receptor confers its high surface expression by JAK2-dependent and -independent mechanisms. J Biol Chem.

